# Long-term effect of uncomplicated *Plasmodium berghei* ANKA malaria on memory and anxiety-like behaviour in C57BL/6 mice

**DOI:** 10.1186/s13071-018-2778-8

**Published:** 2018-03-20

**Authors:** Luciana Pereira de Sousa, Roberto Farina de Almeida, Flávia Lima Ribeiro-Gomes, Leonardo José de Moura Carvalho, Tadeu Mello e Souza, Diogo Onofre Gomes de Souza, Cláudio Tadeu Daniel-Ribeiro

**Affiliations:** 1 Laboratório de Pesquisa em Malária, Instituto Oswaldo Cruz & Centro de Pesquisa, Diagnóstico e Treinamento em Malária (CPD-Mal) da Fundação Oswaldo Cruz (Fiocruz) e da Secretaria de Vigilância em Saúde (SVS), Ministério da Saúde, Rio de Janeiro, Brazil; 2 Instituto de Ciências Básicas da Saúde, Departamento de Bioquímica, Universidade Federal do Rio Grande do Sul, Porto Alegre, Brazil

**Keywords:** *Plasmodium berghei*, Uncomplicated malaria, C57BL/6 mice, Anxiety, Memory, Behavioural impairment

## Abstract

**Background:**

Cerebral malaria, the main complication of *Plasmodium falciparum* infection in humans, is associated with persistent neurocognitive sequels both in human disease and the murine experimental model. In recent years, cognitive deficits related to uncomplicated (non-cerebral) malaria have also been reported in chronically exposed residents of endemic areas, but not in some murine experimental models of non-cerebral malaria. This study aimed at evaluating the influence of uncomplicated malaria on different behavioural paradigms associated with memory and anxiety-like parameters in a murine model that has the ability to develop cerebral malaria.

**Methods:**

*Plasmodium berghei* ANKA-infected and non-infected C57BL/6 mice were used. Development of cerebral malaria was prevented by chloroquine treatment starting on the fourth day of infection. The control group (non-infected mice) were treated with PBS. The effect of uncomplicated malaria infection on locomotor habituation, short and long-term memory and anxious-like behaviour was evaluated 64 days after parasite clearance in assays including open field, object recognition, Y-maze and light/dark tasks.

**Results:**

*Plasmodium berghei* ANKA-infected mice showed significant long-lasting disturbances reflected by a long-term memory-related behaviour on open field and object recognition tasks, accompanied by an anxious-like phenotype availed on open field and light-dark tasks.

**Conclusions:**

Long-term neurocognitive sequels may follow an uncomplicated malaria episode in an experimental model prone to develop cerebral malaria, even if the infection is treated before the appearance of clinical signs of cerebral impairment.

## Background

Malaria, one of the major public health problems in the world, usually manifests with the classic symptoms and signs of fever, sweating, chills and intense headache. It may also result in immunopathological phenomena that accompany the severe forms of the disease [1, 2]. In 1–2% of the cases of infection with *Plasmodium falciparum*, which account for around 90% of the world cases, cerebral malaria (CM), the most severe complication of *falciparum* infection, develops, resulting in about 80% of all the malaria deaths, mainly of children, pregnant women and non-immune adults [3]. Survivors may present neurocognitive sequels, such as behavioural changes, learning deficits and other cognitive impairments, in the short or long-term after the clinical episodes [4–11]. Neurocognitive impairment has also been consistently recorded in murine models of CM [12, 13].

In recent years, poor performance in cognitive events, mainly related to learning and memory, has also been observed in individuals with uncomplicated malaria episodes in different endemic regions of the world [14–18]. They were not, however, observed in some murine experimental models in which CM is not expected to occur [12, 19]. The appearance of CM in some models seems, therefore, to depend on the host-parasite interaction specific to each situation, involving the genetic background of the host and the parasite species and strains and the nature and intensity of the consequent inflammatory response, determining or not damage to brain tissues and vasculature [20].

The late impact of the uncomplicated malaria on cognitive performance is still poorly understood. The present study aimed at searching for a long-lasting influence of uncomplicated experimental murine malaria on different behavioural paradigms, specifically designed to investigate the cognitive performance and anxiety-like phenotype in mice. Considering that the great majority of cases of human malaria due to *P. falciparum* present as non-complicated malaria, we decided to use the classical murine experimental model of CM (*P. berghei* ANKA-infected C57BL/6) treating the mice early (at day 4) in the course of infection to avoid CM development. Our understanding is that this prototype simulates the situation of uncomplicated falciparum malaria, which has the potential of becoming severe and evolving to CM, but will not do so if treated opportunely.

## Methods

### Animals

Seven-week-old C57BL/6 female, mice weighing 20–25 g, were provided by the “Instituto de Ciência e Tecnologia em BioModelos” of the “Fundação Oswaldo Cruz” (ICTB- Fiocruz, Brazil). Mice were kept in cages containing five animals, housed in racks with an air filtration system in a room kept at 25 °C and 12 h light/dark cycles with free access to food and water.

### Infection and treatment of experimental groups

Passage C57BL/6 mice were infected by intraperitoneal (ip) injection with 150 μl of cryopreserved and thawed *Plasmodium berghei* ANKA expressing green fluorescent protein (GFP) (*Pb*A) [21] parasitized red blood cells. Five days after *Pb*A-inoculation, the whole blood of the passage mice was collected, adjusted to 10^6^ parasitized erythrocytes per 100 μl inoculum, and injected by the ip route in C57BL/6 mice of the experimental groups. In this model of infection, the evolution and establishment of CM occurs between the fifth and sixth day [22]. Parasitaemia was determined by flow cytometry, based on the percentage of GFP^+^ erythrocytes. Infected mice were treated on the fourth day of infection, with a mean parasitaemia of 2.5% (range 1.1–4.3) before any clinical sign of CM was apparent. The treatment regimen used [chloroquine (CQ) 25 mg/kg (total dose)] was standardized accordingly to the literature for the treatment of human malaria caused by *Plasmodium* parasites sensitive to this drug [3, 23]. The only difference is that the total dose in mice is given scheduled in 7 days, not 3 (as in humans), since this is the protocol previously show to completely abolish *P. berghei* parasitaemia in mice [12]. *Pb*A infected/treated C57BL/6 mice will be hereafter referred to as infected mice. Phosphate buffered solution (PBS) was administered by gavage to the control (non-infected mice) group that, otherwise, underwent all manipulation procedures to which the experimental group (infected mice) were subjected. Eleven animals were used per group (of infected or non-infected mice) in two rounds of experiments, totalling 22 animals per group.

An experiment aimed at excluding the existence of a possible effect of chloroquine on learning and anxious behaviour was carried out in a group of non-infected mice before the experiments that used treatment in infected mice. Two groups of (twelve animals each) chloroquine treated or non-treated (PBS treated, control) mice were evaluated for behaviour tests and anxiety phenotype, in two rounds of experiments, totalling 24 animals per group.

### Evaluation of behavioural tasks

**Fig. 1:**
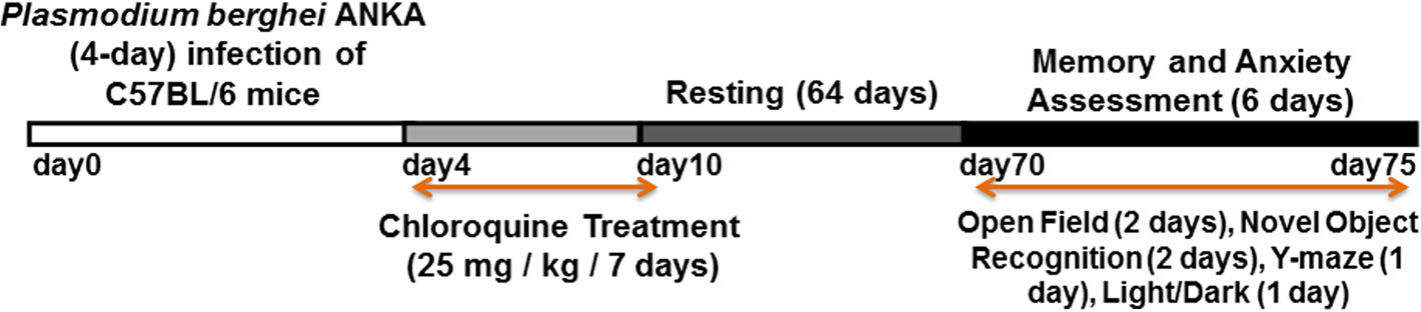
Experiment flowchart. *Plasmodium berghei* ANKA-infected C57BL/6 mice received chloroquine treatment for 7 days, starting on the day 4 of infection. Mice were assessed for performance regarding the indicated behavioural tasks on memory and anxiety-like evaluation 64 days after treatment (resting time)

Sixty-four days after treatment (Fig. 1), animals were evaluated on open field, object recognition, Y-maze and light/dark tasks. Before the sessions, mice were acclimatized in the experimental room for 2 h. All experiments were completed between 2–6 pm and the behavioural performance of mice was analysed using the AnyMaze® software (Stoelting Co., Wood Dale, IL, USA).

#### Behavioural tasks

Both open field and object recognition tasks were evaluated in a grey acrylic box (50 × 50 × 50 cm) with a 200 lux intensity white light. Mice were individually placed and then recorded for 10 min with a video-camera (positioned above and at *c.*90° to the square arena) that was connected to a monitor for further analysis. The apparatus was cleaned with 70% alcohol and dried after all tasks.

#### Open Field task (OFT)

The OFT was used as previously described [24, 25] and was divided into two daily sessions (i.e. 24 h apart), a training (OFT1) and a test session (OFT2), in order to evaluate locomotor activity (total distance travelled in the OFT training and test sessions); habituation to novelty [(i) a significant decrease in the distance travelled during the first to the third minute on the first training session (OFT1) was used to measure short-term habituation to novelty; (ii) a significant decrease in the total distance travelled in the second test session (OFT2), compared to OFT1 was considered a long-term habituation to novelty]; and anxiety-like phenotypes, evaluated by the decrease in time and distance travelled in the centre of the open field arena in the first OFT exposition.

#### Novel Object Recognition task (NORT)

The NORT [26] was used, with minor modifications, to evaluate recognition memory. NORT is based on the tendency of mice to discriminate a novel object (NO) from a familiar object (FO). Two OFT sessions were performed before initiating the NORT protocol in order to reduce the novelty of the arena and decrease the anxiety levels. 24 h after the OFT sessions, the mice were submitted to the NORT training session. Animals were individually placed in the periphery of the apparatus with two identical objects and allowed to explore the arena for 10 min. Object exploration was recorded by an experienced observer only when the animal's nose or mouth was in contact with the objects. During training session both objects were novel and the time spent on both objects should be similar. In the NORT test session, 24 h after the training session, the animals were individually placed back in the arena with one FO (the same as in the previous phase) and one NO in order to measure the long-term memory. The time spent exploring the objects was again recorded for 10 min. The results were expressed as percentage of time exploring each object during the test session. The test session should detect a preference for the NO. Mice that recognized the NO as such and explored it for more than 50% of the total time were considered to display successful recognition.

#### Y-maze task

The Y-maze apparatus consisted of grey wooden walls with 3 identical arms (30 × 8 × 15 cm each at an angle of 120° from the others). The working and short-term memories were evaluated separately using two different protocols and two cohorts of animals. Firstly, the Y-maze task was conducted as described [27] to assess the spontaneous alternation. Mice were placed at the end of one arm and were allowed to explore the maze freely for 8 min without training. An alternation was defined as a complete cycle of consecutive entrances into each of the 3 arms. Percent alternation (PA) was calculated as follows: PA = number of alternations/ (total number of entrances into each arm – 2) × 100. The second approach was a two-trial task with a training phase and a test phase trials [28], separated by an inter trial of 30 min. In the training phase trial, each mouse was individually placed in the maze with one of the 3 arms closed, were allowed freely to explore the 2 arms for 5 min, and then allocated back to the home cage. After 30 min, the animal was again placed in the maze with all 3 arms opened, and was allowed freely to explore all arms. The previously closed arm, opened in the test phase trial, was defined as the new arm. The animal performance was video-recorded for analysis. The time spent and the total distance travelled in the new arm were analysed using AnyMaze® software.

#### Light/Dark task

The light/dark task was performed as previously described [29]. The apparatus consisted of a rectangular acrylic box with two separate chambers of similar dimensions (50 × 50 × 50 cm). One chamber had black walls and floor and was not illuminated. The other side had white walls and floor and was illuminated by a 100W white lamp that was placed overhead. The two compartments were separated by a wall, which had a small opening at the floor level. Each mouse was placed in the light compartment facing away from the opening and allowed to explore the box for 5 min. The following behavioural parameters were analysed by ANY-Maze software: the number of transitions between compartments and the time spent in the light compartment.

### Statistical analysis

The difference in behavioural tasks performance was analysed by a two-way ANOVA test followed by Bonferroni *post-hoc* test to evaluate the effect of different treatments on locomotor activity and short- and long-term memory. Additionally, unpaired Student’s t-test was used to evaluate differences in the anxiety-related behaviours. However, when the sample standard deviation (SD) was different between the groups, the unpaired t-test with Welch’s correction was used; *P* < 0.05 was considered statistically significant.

## Results

To address whether uncomplicated *P. berghei* ANKA (*Pb*A) malaria might influence different behavioural paradigms two months after infection and treatment, C57BL/6 mice were inoculated with 10^6^ parasitized erythrocytes and treated with CQ for seven days from day 4 of infection (Fig. 1).

### Effect of CQ treatment on parasitaemia and on recognition memory or anxiety in mice

**Fig. 2:**
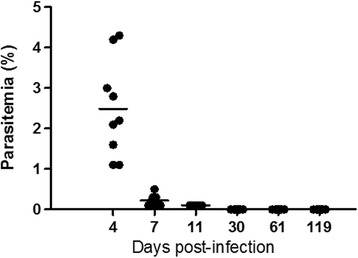
Effect of chloroquine (CQ) administration for full treatment of *Pb*A infection in C57BL/6 mice. CQ was given by gavage at a concentration of 25 mg/kg daily for 7 days (from day 4 to day 10 of infection, *n* = 10, combining 2 experiments). This therapeutic scheme does not allow recrudescence of parasitaemia for up to 4 months of follow-up

**Fig. 3:**
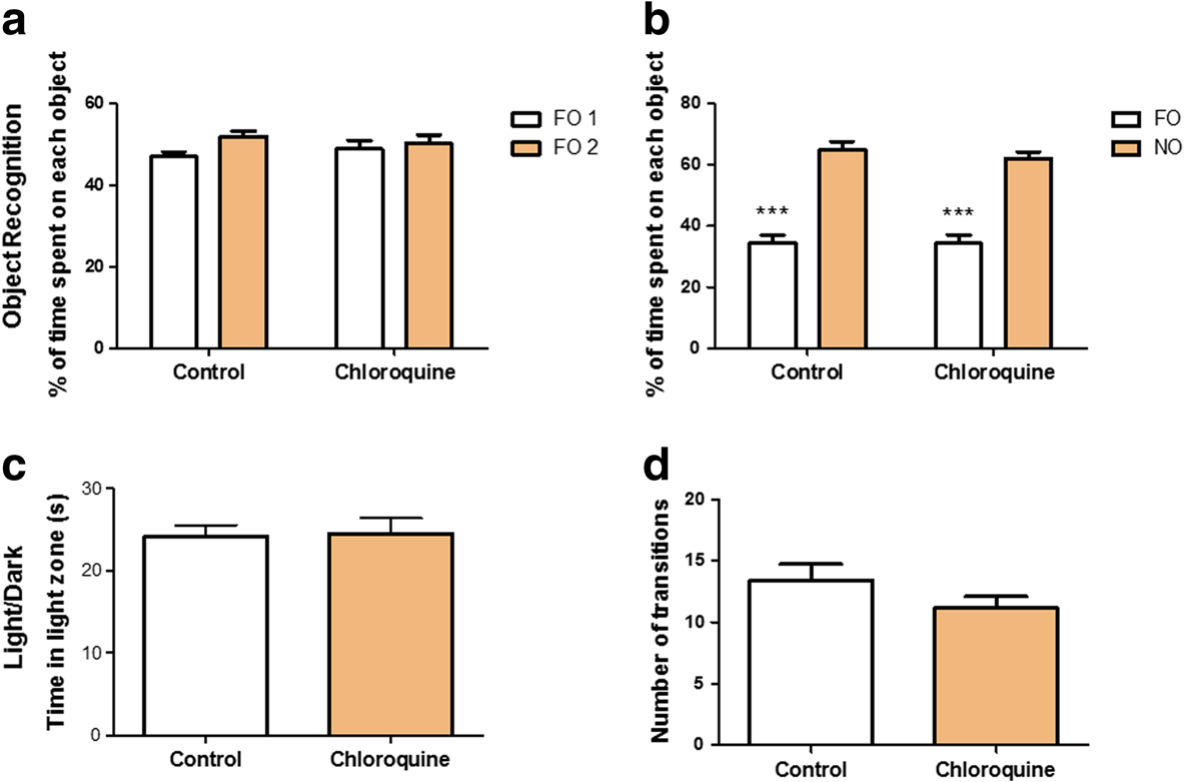
Influence of chloroquine (CQ) administration on recognition memory and anxiety-like parameters in non-infected animals. CQ was used by gavage at a concentration of 25 mg/kg daily for 7 days (from day 4 to day 10 of infection, *n* = 10, combining 2 experiments). This therapeutic scheme does not affect the recognition memory (training **a**; test **b**) and the anxiety-like parameters (**c** and **d**; *n* = 24, combining 2 experiments) performed 64 days after the administration of CQ on the NO Recognition and Light/Dark task. Control: PBS treated mice. *Abbreviations*: FO, familiar object; NO, novel object

None of the animals treated with CQ for 7 days showed recrudescence of parasitaemia for up to 4 months of follow-up (Fig. 2) and this therapeutic scheme alone did not influence either the recognition memory (ANOVA: *F*_(1, 52)_ = 1.390, *P* = 0.759, Fig. 3a, b) or the anxiety-like parameters (t-test: *t*_(26)_ = 0.1517, *P* = 0.8806, Fig. 3c, d), the two important and central behavioural features explored in detail in this study.

### Effect of uncomplicated malaria on locomotor activity

**Fig. 4:**
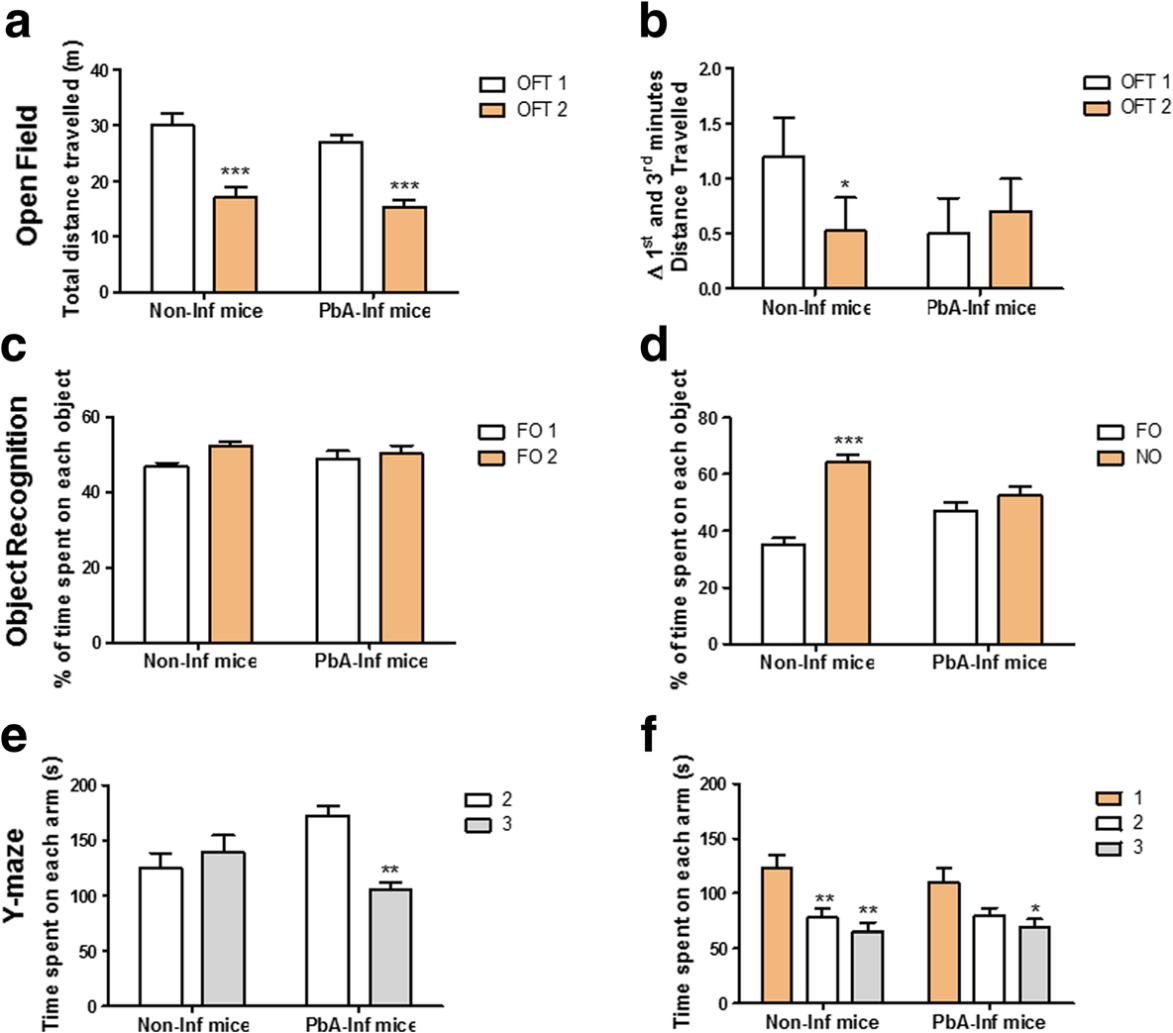
*Pb*A-infected mice show long-lasting impairment in memory-related task performance. Effect of a treated *Pb*A 4 day-infection on the total distance travelled in open field training and test sessions (**a**) and on the Δ between the first and the third minute distance travelled (**b**); effect of *Pb*A 4 day-infection on the time spent in each object in the training (**c**) and test (**d**) sessions on the novel object recognition task; and effect of treated *Pb*A 4 day-infection on time spent in each arm in the training (**e**) and test (**f**) sessions in the Y-Maze task. Data were analysed by two-way ANOVA, followed by Tukey’s multiple range *post-hoc* test. *P < 0.05, **P < 0.01 and ***P < 0.001, comparing the training (OFT1) and test (OFT2) sessions; the familiar (FO) and the novel (NO) object recognition task; and the different arms in the Y-maze task (*n* = 22 animals/group, combining 2 experiments). See Methods for behavioural task description. Arm 1 (closed and open on training and test sessions, respectively), arms 2 and 3 (open). *Abbreviations*: Non-inf mice, uninfected and PBS treated mice; *Pb*A-inf mice, infected and chloroquine treated mice

*Pb*A infected/treated mice showed no difference in the total distance travelled in both OFTs paradigms in relation to the control group (both groups; ANOVA: *F*_(1, 80)_ = 52.36, *P* **<** 0.0001, Fig. 4a).

### Effect of uncomplicated malaria on different memory-related behaviours

Both infected and non-infected groups significantly decreased the total distance travelled across the OFT test sessions, an important index of long-term habituation memory (ANOVA: *F*_(1, 80)_ = 52.36, *P* **<** 0.0001, Fig. 4a). However, the *Pb*A-infected mice presented no change in the distance travelled between the first and third minute of OFT training and test session (ANOVA: *F*_(1, 80)_ = 0.2301, *P* = 0.5365), as compared to non-infected mice in which distance travelled decreased in OFT2 (ANOVA: *F*_(1, 80)_ = 4.88, *P* = 0.0392, Fig. 4b), suggesting a short-term habituation to novelty impairment.

No significant difference in the time spent on each familiar object was observed in the Novel Object Recognition task (NORT) training session between non-infected and infected groups (ANOVA: *F*_(1, 77)_ = 1.088, *P* = 0.3002; *F*_(1, 80)_ = 0.01834, *P* = 0.8926, Fig. 4c). In the test session, however, only non-infected mice recognized the NO (long-term recognition memory) since their NO exploration time was markedly higher than the FO exploration time (ANOVA: *F*_(1, 80)_ = 35.51, *P* = 0.0009, Fig. 4d). *Pb*A-infected mice showed no difference between NO and FO exploration time (ANOVA: *F*_(1, 80)_ = 0.431, *P* = 0.5281, Fig. 4d).

Moreover, a significant increase was observed in time spent in the arm at which infected mice were initially placed (arm 2) in the training session of the Y-maze task (ANOVA: *F*_(1, 32)_ = 10.44, *P* = 0.0024, Fig. 4e) and a different temporal pattern of exploration during testing was registered, comparing the new arm (arm 1) with the other two arms (arms 2 and 3) (ANOVA: *F*_(2, 48)_ = 4.899, *P* = 0.0136 and *F*_(2, 48)_ = 6.283, *P* = 0.0007 in control mice and *F*_(2, 48)_ = 2.196, *P* = 0.1260 and *F*_(2, 48)_ = 4.899, *P* = 0.0194 in the *Pb*A infected mice; Fig. 4f). No difference in the percentage of spontaneous arm alternance was observed between *Pb*A-infected and non-infected mice in spatial working memory in the Y-maze task (data not shown).

### Effect of uncomplicated malaria on anxiety

**Fig. 5:**
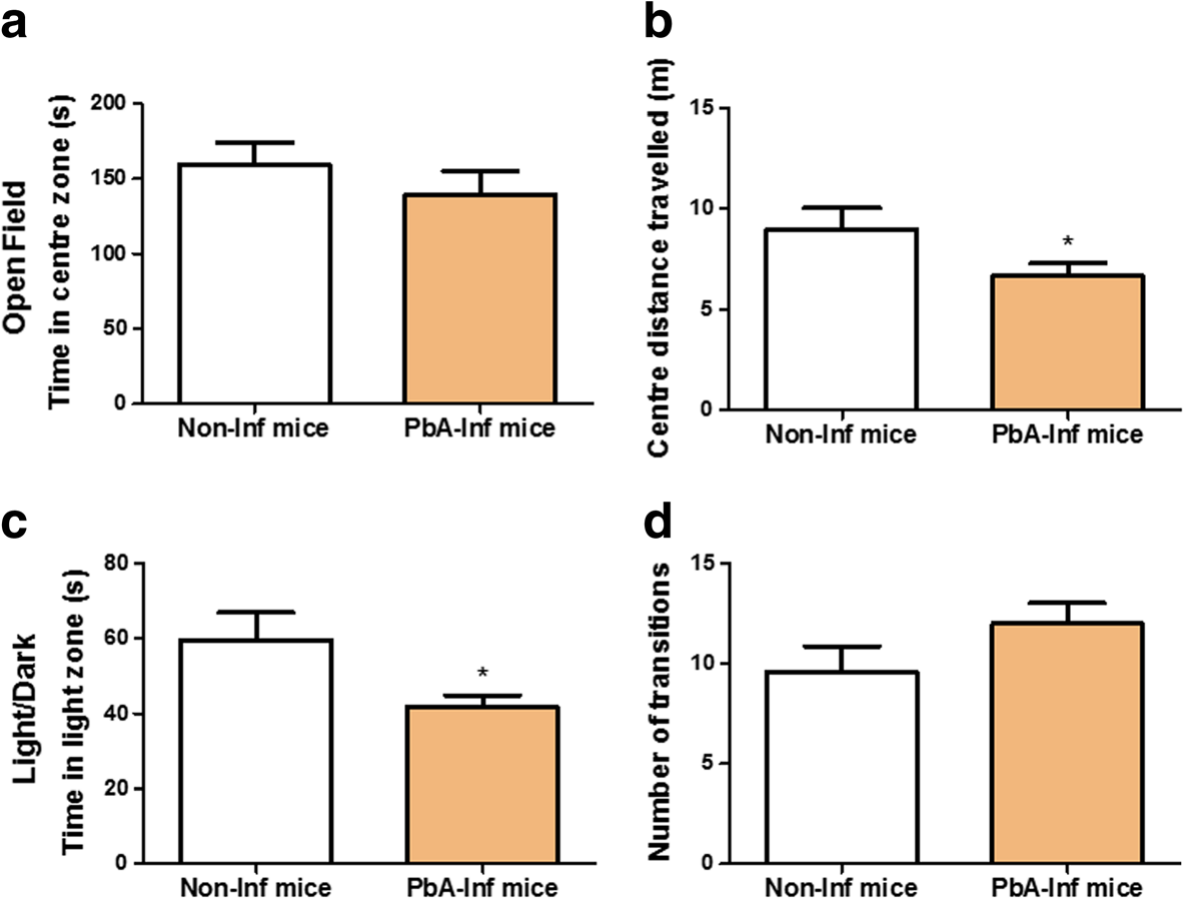
*Pb*A-infected mice presented long-lasting increase in anxious-related behaviour. The distance travelled (**a**) in the centre zone was evaluated in the open field task training session (*n* = 22, combining 2 experiments). The time spent in the light compartment (**b**) was evaluated in the light/dark task (*n* = 12). Data are reported as the mean ± SEM and were analysed by unpaired Student’s t-test. *P < 0.05 compared to the non-infected animals. See Methods for behavioural task description. *Abbreviations*: Non-inf mice, uninfected and PBS treated mice; *Pb*A-inf mice, infected and chloroquine treated mice

A significant decrease in the distance travelled in the centre zone during OFT1 (t-test: *t*_(40)_ = 2.705, *P* = 0.0284, Fig. 5b) and a significant reduction in time spent in the light zone in the light/dark task (t-test: *t*_(16)_ = 2.089, *P* = 0.0415, Fig. 5c) was observed in *PbA* infected mice. No difference in time in the centre zone in OFT1 (t-test: *t*_(40)_ = 0.9658, *P* = 0.3580) and number of transitions in light/dark task (t-test: *t*_(16)_ = 1.423, *P* = 0.1149, Fig. 5a, d) was observed in these animals.

## Discussion

The results described here are, to our knowledge, the first to present robust evidence of a long-term cognitive impairment in murine experimental uncomplicated malaria. *Pb*A-infected C57BL/6 mice exhibited impairment of the recognition memory as well as an increase in the anxiety-like phenotype 64 days after parasite clearance and 71 days after a four days long-infection.

The novel object recognition task (NORT) is an excellent tool to measure parameters related to recognition memory in rodents. Data obtained with this test indicate that the long-term memory of *Pb*A infected mice was impaired even when tested 64 days after treatment. In agreement, deficits in long-term memory after experimental CM have been described both during infection [30–32] and 30–40 days after treatment [12, 13]. However, cognitive deficits were not observed in various murine experimental models of non-CM such as *Pb*A-infected BALB/c mice, *P. chabaudi chabaudi-*infected and *P. chabaudi adami-*infected C57BL/6 and 17NL *Plasmodium yoelii*-infected Swiss mice [12, 19]. It must be said that the host-parasite interplay can modulate the pathogenesis of the experimental malaria determining the disease outcome. For example, the profile of immune response to *Pb*A differs according to the mice genetic background, being more inflammatory (Th1 profile) in C57BL/6 mice, than in BALB/c mice (Th2 profile) infected with the same parasite, thus resulting in susceptibility (C57BL/6) or resistance (BALB/c), to the development of CM and, possibly, the emergence or not of neurocognitive dysfunctions [33].

Important features related to cognitive performance, such as short- and long-term habituation to novelty (a parameter strictly related with spatial memory), have been measured in the OFT. Usually, when mice assess for the first time the open field arena, a high rate of locomotor activity is observed. However, over time (normally up to 3 min) this exploratory behaviour decreases significantly, since the stress related to novelty disappears (short-term habituation) [25, 34–42]. Similarly, when mice re-assess the familiar open field arena, they normally show a slower exploratory behaviour called long-term habituation [23]. The results of OFT displayed here may suggest a short-term habituation to novelty impairment in *Pb*A-infected mice. We did not observe any disturbance in total distance travelled comparing *Pb*A-infected mice with non-infected mice in the OFT.

The differential effects of long-term memory for either object recognition (Fig. 4d) or spatial habituation (Fig. 4a) are not surprising, since previous studies have shown that long-term habituation is more robust than other memories, such as that for inhibitory avoidance task [43]. Memory habituation is impaired in surviving CM mice 40 days after treatment [12]. However, a similar study, conducted in a model of uncomplicated experimental malaria (*Plasmodium chabaudi adami*-infected C57BL/6 mice) [19], evaluated the locomotor performance 15 days after parasite clearance and observed no difference in total distance covered or velocity between infected and control mice. Moreover, CM (*Pb*A-infected C57BL/6) surviving mice did not present any change in locomotor behaviour, although they showed a significant motor deficit 40 days after CQ treatment [13]. These data suggest that the motor impairment is strictly related to severe malaria.

In spite of the recorded impairment in the short-term habituation in OFT1, no long-lasting effect of uncomplicated *Pb*A infection was observed on the Y-maze task, including the specific parameter used to analyze the working memory (percentage of spontaneous arm alternance). On the other hand, the study provides evidence that indicates that *Pb*A-infected mice present long-lasting anxiety-like behaviour, since it was observed a significant decrease in the distance travelled in the centre zone during OFT1, reduced time in the light zone in the light/dark task, and increased time in the arm 2 where they were initially placed in the Y-maze, including a different OFT1 temporal pattern of exploration, mainly in the first minutes of the task. Actually, anxious animals prefer not to be exposed to situations in which they may be exposed to predators, such as an open, novel or light environment. Taken together, these results seem to indicate that the initial evidence concerning the impairment in short-term habituation to novelty may be not due to short-term cognitive deficits but rather to an increase in the anxious-like behaviour.

Increase in anxiety-like behaviour was previously reported in an uncomplicated malaria experimental model (*Plasmodium chabaudi adami*-infected C57BL/6 mice), 15 days after parasite clearance [19]. Anxious behaviour was also detected, at the high cross-maze [31] and the open field behaviour [32] tasks, at day 5 of infection in the same model of the present study (*Pb*A-infected C57BL/6 mice). However, one must keep in mind that on day 5 when the behaviour was assessed, animals are usually sick (feverish and toxemic) and progressing to or already presenting CM.

Considering the results reported here, we can hypothesize that even a short-term *Pb*A-infection, treated before the appearance of any clinical sign of neurological impairment in C57BL/6 mice [44], a model susceptible to experimental CM, may result in long-term memory deficits and anxiety-like behaviour. Studying this same model, another author has shown that as early as at the fourth day of infection, the leukocyte adhesion process in the cerebral microvasculature had already started, even in the absence of petechial haemorrhage signs and with minimal formation of oedema [22]. This precedes the evident and typical neuropathological CM signs starting on day 5 of infection, with the usual full establishment on day 6 of infection in the model studied and could explain, at least partially, the results reported here. However, leukocyte accumulation has been also shown to occur in the non-CM model of *P. berghei* NK65 infection of C57BL/6 mice [45], and, moreover, preliminary data shows that no blood-brain barrier breakdown is observed on day 4 in the *Pb*A-C57BL/6 model studied here (unpublished observations).

In this study, the behavioural effects of malaria in C57BL/6 PbA-infected mice were recorded late (70 days after the infection) in animals treated, at day 4, before any clinical manifestation suggestive of brain tissue damage. This late observation of long-term cognitive deficits as sequels of uncomplicated malaria in a CM-prone host-parasite model may be considered as an informative example of the potential situation in human falciparum malaria.

As far as pathogenesis of the observed behavioural effects is concerned, some mechanisms have been proposed to explain behavioural changes in the absence of neural injury, including hypoxia, depression, attention deficit, anxiety, malnutrition, medications and normal aging [46]. We can, therefore, ponder that in the early phases of the infectious process establishment, when minimal changes in the brain physiology and structure may be starting (as the slight leukocyte adhesion and minimum oedema in the cerebral microvasculature, in the absence of petechial haemorrhage recorded at day 4 by Potter et al. [22]), accompanied by the emerging inflammatory environment and probably disturbances in the glial cells homeostasis (directly) and the triggering of an anxious behavioural (indirectly) could influence the cognitive performance of the experimental mice.

Finally, we can speculate that the equivalent time in humans of manifestation of the memory impairment and anxiety-like phenotypes, observed here in mice, based on longevity parameters [47] suggests that cognitive impairments could be detected 7 years after an episode of mild malaria in a human.

## Conclusions

Long-lasting cognitive impairments, related to learning and memory, have never been reported as sequels of mild malaria in murine models. Here, evidence of short- and long-term memory deficits 64 days after parasite clearance by CQ treatment of *Pb*A-infected C57BL/6 mice is presented for the first time. Further research in other strains of mice susceptible or insusceptible to develop CM is needed to confirm these data and to elucidate the dynamics of cognitive deficits and anxious related parameters in uncomplicated malaria.

## Abbreviations

CM: cerebral malaria
CQ: chloroquine
*Pb*A: *Plasmodium berghei* ANKA
OFT: Open Field task
NORT: Novel Object Recognition task
GFP: green fluorescent protein
WHO: World Health Organization
